# Valection: design optimization for validation and verification studies

**DOI:** 10.1186/s12859-018-2391-z

**Published:** 2018-09-25

**Authors:** Christopher I Cooper, Delia Yao, Dorota H Sendorek, Takafumi N Yamaguchi, Christine P’ng, Kathleen E Houlahan, Cristian Caloian, Michael Fraser, SMC-DNA Challenge Participants, Kyle Ellrott, Adam A Margolin, Robert G Bristow, Joshua M Stuart, Paul C Boutros

**Affiliations:** 1 Ontario Institute for Cancer Research, 661 University Avenue, Suite 510, Toronto, Ontario M5G 0A3 Canada; 2 Department of Medical Biophysics, University of Toronto, Toronto, Canada; 3 Princess Margaret Cancer Centre, University Health Network, Toronto, Canada; 4 Computational Biology Program, Oregon Health & Science University, Portland, OR USA; 5 Department of Biomedical Engineering, Oregon Health & Science University, Portland, OR USA; 6 Department of Biomolecular Engineering, University of California Santa Cruz, Santa Cruz, CA USA; 7 Sage Bionetworks, Seattle, WA USA; 8 Department of Pharmacology & Toxicology, University of Toronto, Toronto, Canada; 9 Departments of Human Genetics & Urology, University of California, Los Angeles, USA; 10 Jonsson Comprehensive Cancer Centre, University of California, Los Angeles, USA; 11 Institute for Precision Health, University of California, Los Angeles, USA

**Keywords:** Verification, Validation, Candidate-selection, DNA sequencing

## Abstract

**Background:**

Platform-specific error profiles necessitate confirmatory studies where predictions made on data generated using one technology are additionally verified by processing the same samples on an orthogonal technology. However, verifying all predictions can be costly and redundant, and testing a subset of findings is often used to estimate the true error profile.

**Results:**

To determine how to create subsets of predictions for validation that maximize accuracy of global error profile inference, we developed Valection, a software program that implements multiple strategies for the selection of verification candidates. We evaluated these selection strategies on one simulated and two experimental datasets.

**Conclusions:**

Valection is implemented in multiple programming languages, available at: http://labs.oicr.on.ca/boutros-lab/software/valection

**Electronic supplementary material:**

The online version of this article (10.1186/s12859-018-2391-z) contains supplementary material, which is available to authorized users.

## Background

High-throughput genomics studies often exhibit error profiles that are biased towards certain data characteristics. For example, predictions of single-nucleotide variants (SNVs) from DNA sequencing data have error profiles biased by local sequence context [1, 2], mappability of the region [3] and many other factors [4, 5]. The false positive rate for individual predictions in high-throughput studies can be high [6, 7], while the false negative rate is difficult to estimate and rarely known. Critically, error rates can vary significantly between studies because of tissue-specific characteristics, such as DNA quality and sample purity, and differences in data processing pipelines and analytical tools. In cancer studies, variations in normal tissue contamination can further confound genomic and transcriptomic analyses [8–10].

Taken together, these factors have necessitated the wide-spread use of studies with orthogonal technologies, both to verify key hits of interest and to quantify the global error rate of specific pipelines. In contrast to a *validation study*, which typically approaches the same biological question using an independent set of samples (e.g. like a test dataset in a machine learning exercise), we define a *verification study* as interrogating the same sample-set with an independent method (e.g. a method that generates analogous data using a distinct chemistry). The underlying concept is that if the second technique has separate error profiles from the first, a comparative analysis can readily identify false positives (e.g. in inconsistent, low quality calls) and even begin to elucidate the false negative rate (e.g. from discordant, high quality calls).

The choice of verification platform is critical as it determines both the tissue and financial resources required. There is typically a wide range of potential verification technologies for any given study. While confirmation of DNA-sequencing results traditionally involves gold-standard Sanger sequencing [11, 12], the drawbacks of this approach (e.g. high financial and resource costs) and advancements in newer sequencing techniques have shifted the burden of variant verification to other technologies [13–15]. For example, a typical Illumina-based next-generation sequencing (NGS) whole-genome or whole-exome experiment may be verified by sequencing a separate library on a different but similar machine [16]. This offers the advantages of high-throughput, low cost and the opportunity to interrogate inter-library differences [17]. Other groups have applied mass-spectrometric based corroboration of individual variants, which has the benefit of technological independence [18, 19].

Apart from choice of technology, all groups must make decisions regarding the *scope* of their verification work. For example when considering genome-wide discovery, it may be appropriate to verify only known candidate drug target mutations or unexpected novel functional aberrations. However, in many contexts having an unbiased estimate of the global error rate is critical. This is particularly true when benchmarking different data-generating methods or when looking at genome-wide trends. It remains unclear how best to select targets for verification studies, particularly in the context of fairly comparing multiple methods and providing unbiased performance metric estimates. To address this problem, we created Valection, a software tool that implements a series of diverse variable selection strategies, thereby providing the first framework for guiding optimal selection of verification candidates. To benchmark different strategies, we exploit data from the ICGC-TCGA DREAM Somatic Mutation Calling Challenge (SMC-DNA), where we have a total of 2,051,714 predictions of somatic SNVs made by 21 teams through 261 analyses [4, 20]. The advantage of these simulated data are that truth is fully known, allowing analysis of both false positive and false negative rates. Additionally, we evaluated Valection selection strategies on two experimental datasets: seven sets of single-nucleotide polymorphisms (SNP) from the Genome in a Bottle (GIAB) Consortium [21, 22] and 15 sets of somatic SNVs from a chronic lymphocytic leukaemia (CLL) tumour-normal pair [23]. We show that the optimal strategy changes in a predictable way based on characteristics of the verification experiments.

## Implementation

**Fig. 1:**
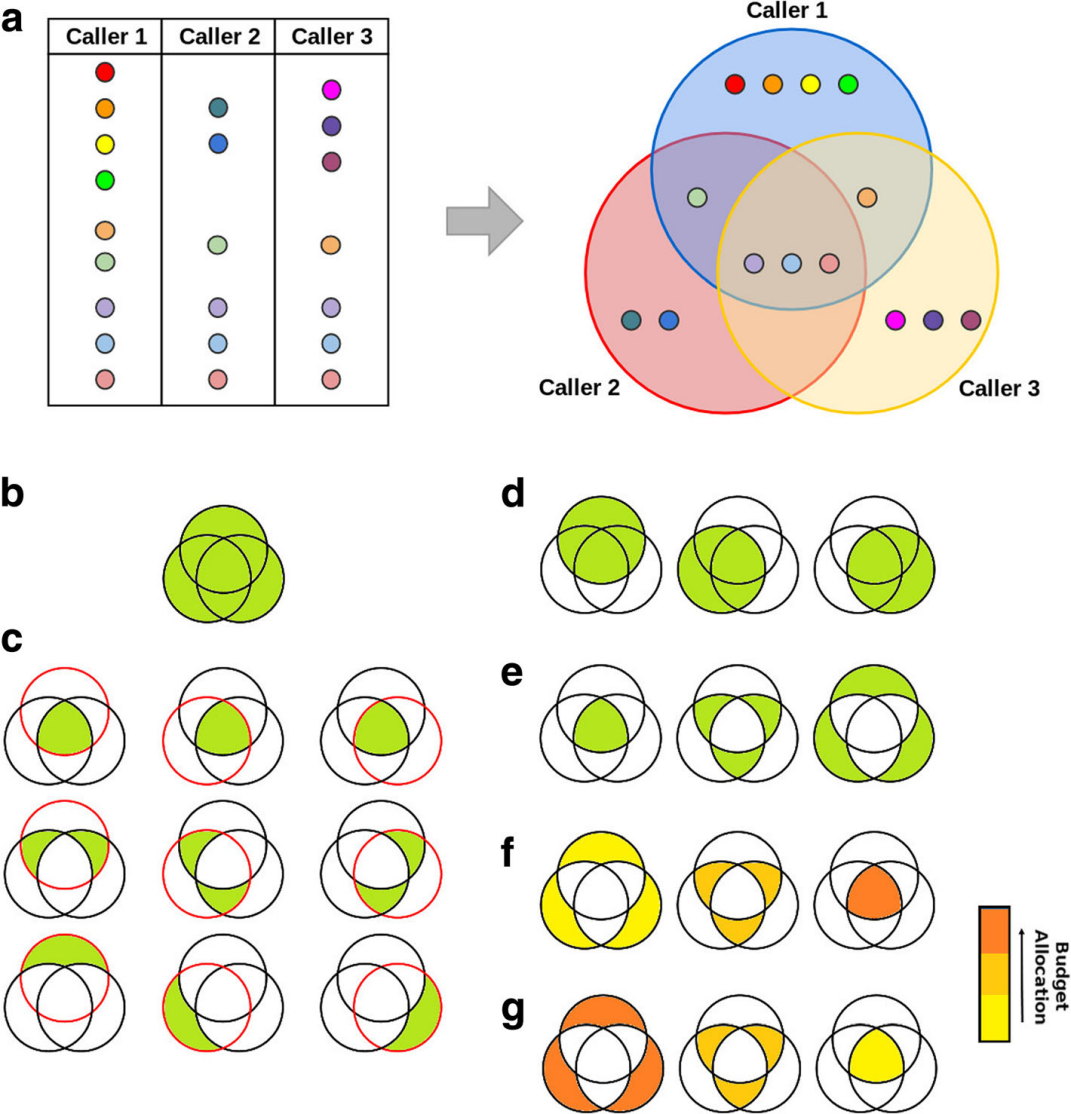
Valection Candidate-Selection Strategies. **a** A hypothetical scenario where we have results from three callers available. Each call is represented using a dot. SNV calls that are shared by multiple callers are represented with matching dot colours. **b** The ‘random rows’ method where all unique calls across all callers are sampled from with equal probability. **c** The ‘directed-sampling’ method where a ‘call overlap-by-caller’ matrix is constructed and the selection budget is distributed equally across all cells. **d** The ‘equal per caller’ method where the selection budget is distributed evenly across all callers. **e** The ‘equal per overlap’ method where the selection budget is distributed evenly across all levels of overlap (i.e. call recurrence across callers). **f** The ‘increasing with overlap’ method where the selection budget is distributed across overlap levels in proportion to the level of overlap. **g** The ‘decreasing with overlap’ method where the selection budget is distributed across overlap levels in inverse proportion to the level of overlap

We began by developing six separate strategies for selecting candidates for verification (Fig. 1). The first is a naïve approach that samples each mutation with equal probability, independent of whether a mutation is predicted by multiple algorithms or of how many calls a given algorithm has made (‘random rows’). Two simple approaches follow that divide mutations either by recurrence (‘equal per overlap’) or by which algorithm made the call (‘equal per caller’). Finally, we created three approaches that account for both factors: ‘increasing per overlap’ (where the probability of selection increases with call recurrence), ‘decreasing per overlap’ (where the probability of selection decreases with call recurrence) and ‘directed-sampling’ (where the probability of selection increases with call recurrence while ensuring an equal proportion of targets is selected from each caller). All methods have programmatic bindings in four separate open-source languages (C, R, Perl and Python) and are accessible through a systematic API through the Valection software package. Valection thus becomes a test-bed for groups to try new ways of optimizing verification candidate-selection strategies.

**Fig. 2:**
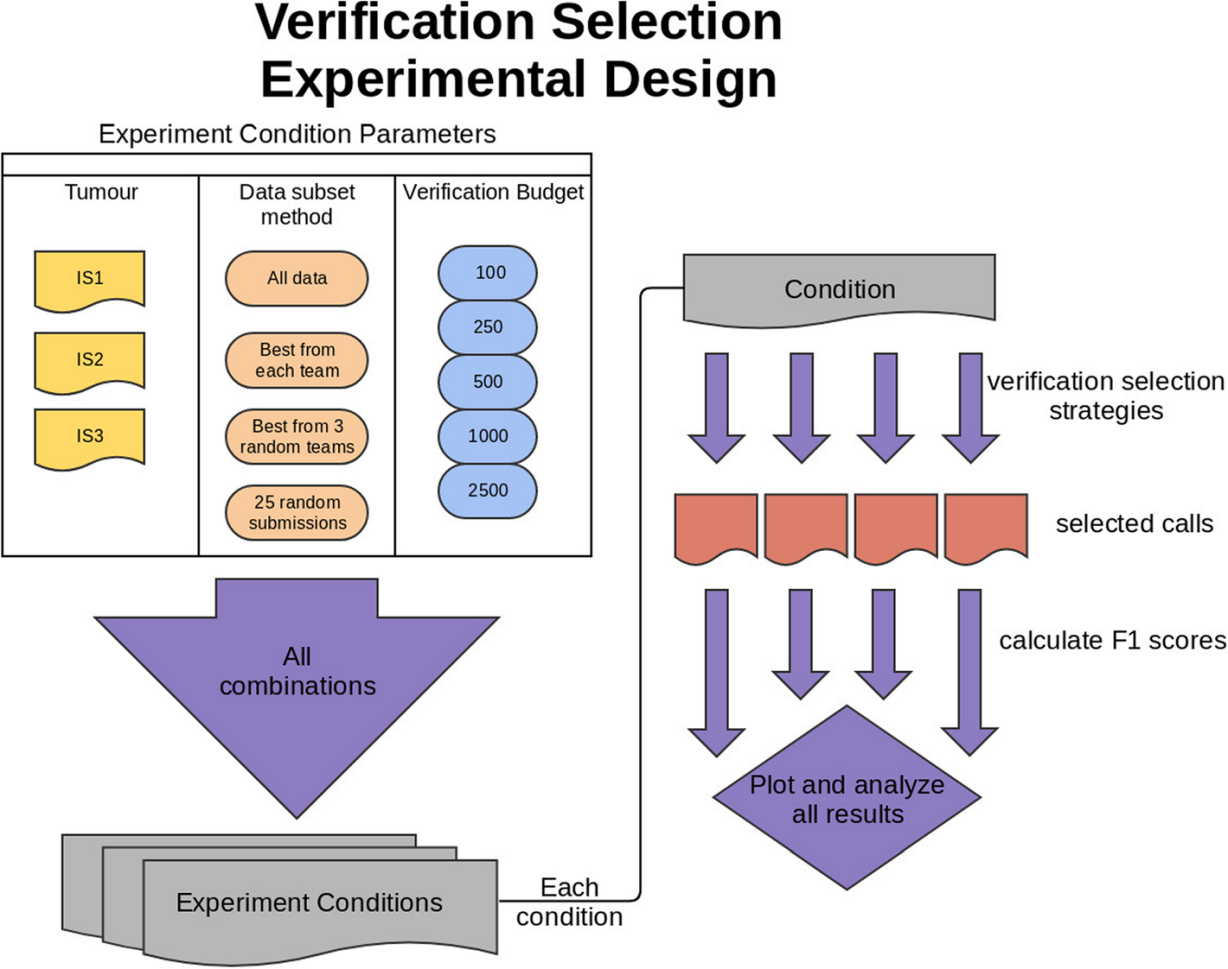
Verification Selection Experimental Design. Verification candidates were selected from somatic mutation calling results of multiple algorithms run on three in silico tumours (IS1, IS2, and IS3). Candidate selection was performed separately on each tumour’s set of results using all combinations of five different verification budgets (i.e. number of calls selected) and six different selection strategies. F_1_ scores were calculated for each set of selected calls and compared to F_1_ scores calculated from the full prediction set. To compare the effect of the numbers of algorithms used, datasets were further subset using four different metrics

To compare the six methods outlined above, we used data from tumour-normal whole-genome sequencing pairs from the ICGC-TCGA DREAM Somatic Mutation Calling Challenge [4, 20]. These tumours differ in major characteristics such as normal contamination, sub-clonality and mutation rate. We chose to work with simulated tumours because we know the ground truth of their mutational profiles, allowing a precise evaluation of the effectiveness of different selection schemes in estimating the true underlying error rates. Altogether, there are results available from 261 SNV calling analyses performed by 21 teams. We designed a rigorous parameter-sweeping strategy, considering different numbers of SNV calling algorithms and different quantities of verification candidate targets. The experimental design is outlined in Fig. 2.

## Results

We assessed the performance of the candidate-selection strategies in two ways. First, we considered how close the predicted F_1_ score from a simulated verification experiment is to that from the overall study. We calculated precision in two modes: ‘default’ (as described in Methods) and ‘weighted’. In the ‘weighted’ mode, precision scores are modified so that unique calls carry more weight than calls predicted by multiple callers. This places more emphasis on true positive calls that are unique to a single submission (i.e. SNVs that are more difficult to detect) over those that are found across multiple submissions. This is important to consider, given that one key goal of SNV calling is to maximize the number of true mutations detected. Second, we assessed the variability in this result across 10 replicate runs of each strategy, allowing us to gauge how much random chance elements of variant-selection perturb the results of a given method (i.e. a stability analysis).

**Fig. 3:**
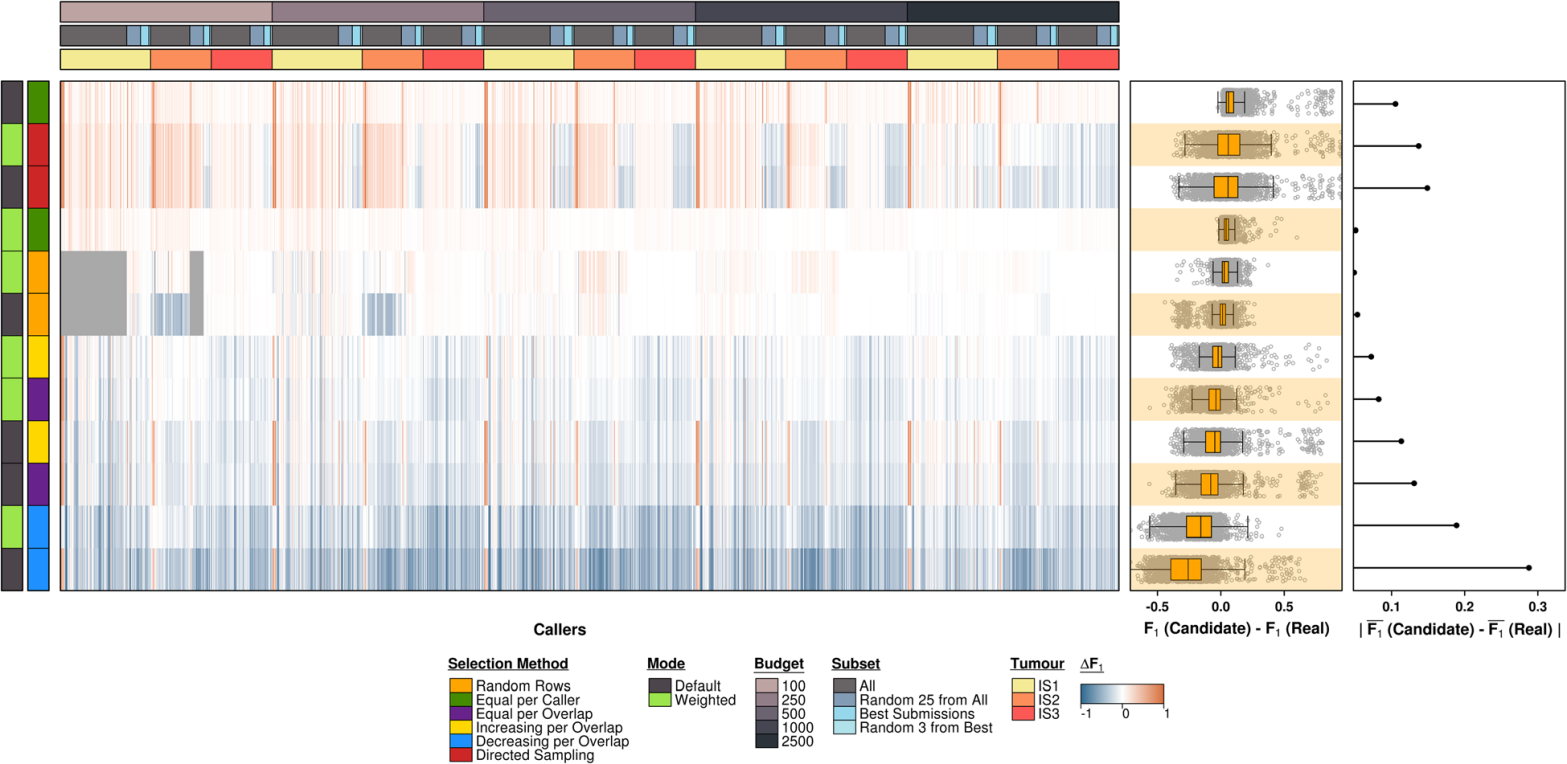
All Synthetic Data Simulation Results for Selection Strategy Parameter Combinations. Overall, the best results are obtained using the ‘equal per caller’ method. The ‘random rows’ approach scores comparably except in cases where there is high variability in prediction set sizes across callers. Calls from low-call callers are less likely to be sampled at random and, in cases where none are sampled, it is not possible to get performance estimates for those callers. Failed estimate runs are displayed in grey

Overall, across all simulations, the ‘equal per caller’ approach performs best, showing a negligible mean difference between subset and total F_1_ scores while, additionally, displaying low variability (i.e. small spread) in F_1_ score differences across all runs (Fig. 3). Both the number of algorithms tested and the verification budget size (i.e. the number of candidates being selected) factor into which strategy performs optimally. Specifically, when there are large numbers of algorithms or the number of possible verification targets is low, the ‘equal per caller’ method does extremely well (n_targets_ = 100; Additional file 1: Figure S1). By contrast, when the number of verification targets is substantially larger (i.e. a considerable proportion of all predictions will be tested), the ‘random rows’ method shows similar performance levels (n_targets_ = 1000 and n_targets_ = 2500; Additional file 1: Figures S2 and S3, respectively). However, the ‘random rows’ method performs poorly when prediction set sizes are highly variable (i.e. a small number of callers has a large fraction of the total calls), resulting in some callers with no calls by which to estimate performance. This was the case for runs with verification budgets of n_targets_ = 250 (Additional file 1: Figure S4), n_targets_ = 500 (Additional file 1: Figure S5) and, in particular, n_targets_ = 100 (Additional file 1: Figure S1). Missing scores were treated as missing data**.**

However, the effects of the verification experiment characteristics described above alone do not account for all the variability observed across the simulations. Comparing runs of matching parameter combinations across the three synthetic tumours reveals some inter-tumour differences. Unlike with tumours IS1 (Additional file 1: Figure S6) and IS2 (Additional file 1: Figure S7), the ‘random rows’ method performs best on tumour IS3 suggesting tumour characteristics may have an impact on target selection strategy performance (Additional file 1: Figure S8). The ‘equal per caller’ method is only the second best selection strategy for the IS3 dataset.

**Fig. 4:**
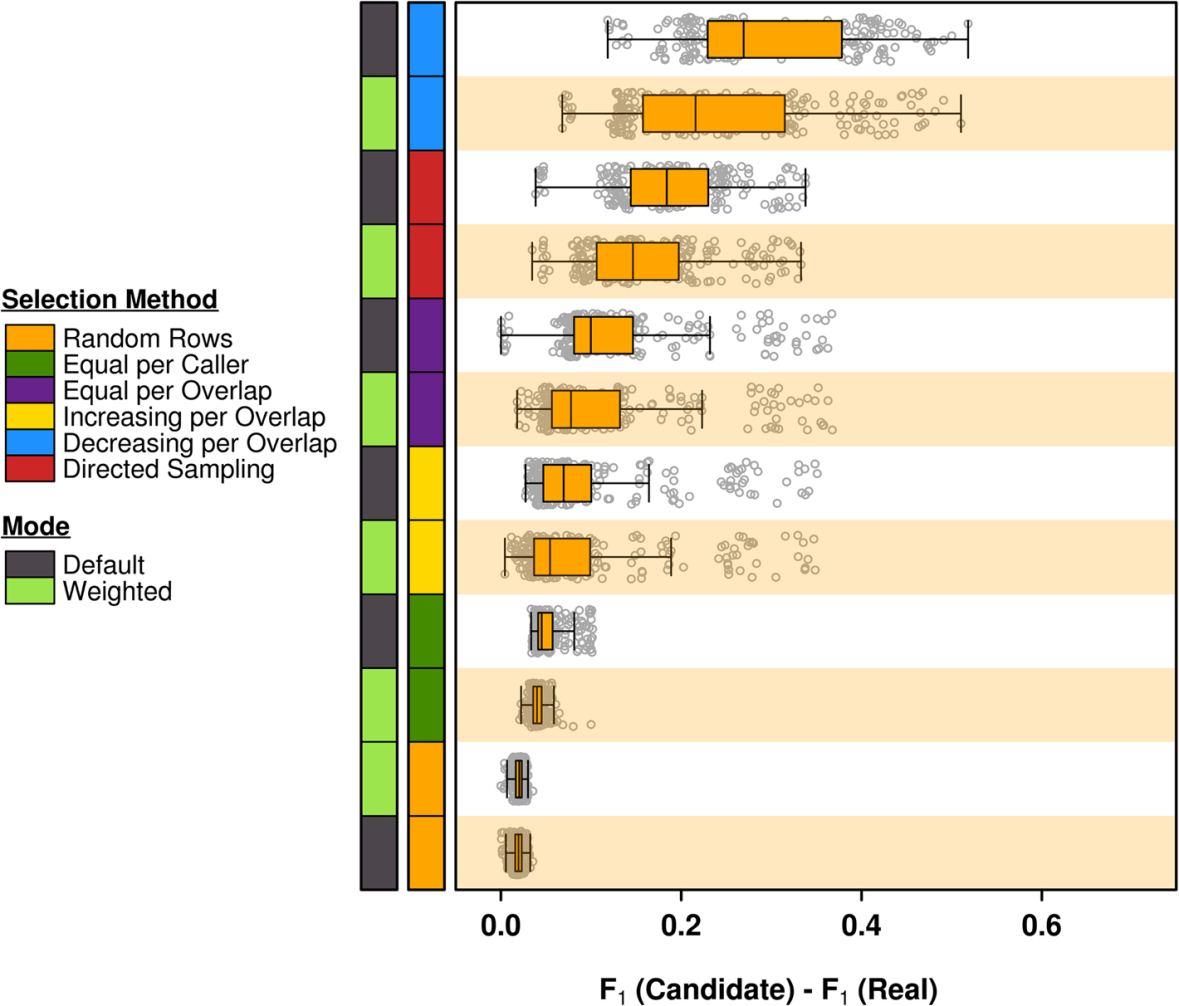
F_1_ Scores for All Synthetic Dataset Replicate Runs. Top selection strategies perform consistently across replicate runs. Strategies are ordered by median scores. The adjustment step in precision calculations improves the ‘equal per caller’ method, but shows little effect on ‘random rows’

We further assessed variability in the results of the selection strategies by running 10 replicate runs of each. The results in Fig. 4 show that the consistency of performance across simulations trends with the overall performance of the selection strategy. An overall positive effect of the adjustment step (‘weighted mode’) on the selection strategies is also visible with the exception of the ‘random rows’ method, on which the weighted precision calculation appears to have no effect. A closer look at the recall and precision scores reveals that the approach with the poorest recall score, ‘decreasing with overlap’ (Additional file 1: Figure S9a), also shows the most sensitivity to the weighted adjustment step in precision calculations (Additional file 1: Figure S9b). Altogether, across methods, recall tended to mirror F_1_ in both magnitude and amount of spread, which is lower in approaches with higher recall. In contrast, precision scores are highly variable across most selection approaches, regardless of their overall performance.

Additionally, we looked at the effect that the number of call sets sampled from has on selection strategy rankings. We performed two comparisons: a) using the complete submission set (all submissions versus a subset of 25 randomly selected submissions per tumour) and b) using only the best team submissions per tumour (all submissions versus a subset of 3 randomly selected submissions). For each comparison group, scores were calculated as before. When selection strategies are ranked by median differences, we see that the ‘random rows’ method most consistently appears in the top performance ranks among all submission sets (Additional file 1: Figures S10 and S11). The ‘decreasing per overlap’ method using default precision calculations is always the worst performing selection strategy, followed by ‘decreasing per overlap’ with weighted precision scores. The performance rankings of the other selection strategies are more variable across submission sets.

While simulated data has fully known truth and thus allows precise analysis of false-positive and false-negative rates, it also represents only a subset of experimental scenarios therefore we assessed the Valection selection strategies on real data by enlisting two separate experimental datasets. First, we evaluated on the germline SNPs in sample NA12878 of the GIAB Consortium, whose genome has been extensively characterized by combining information from various sequencing technologies and bioinformatics pipelines [21, 22]. We collected seven publicly-available VCF files containing germline variant calls obtained from NA12878 DNA samples that were processed on one of five different sequencing platforms, using one of four variant calling methods (NIST v3.3.2). Integrated, high-confidence SNP calls provided by the consortium in the same data release served as the mutational ground truth for our analysis. Results reveal the ‘random rows’ method as the top selection strategy in terms of overall highest mean performance as well as performance consistency (Additional file 1: Figure S12), which is consistent with the strategy’s high ranking in the simulated tumour analysis. In addition to running the evaluation at the original synthetic data candidate budget sizes, we ran Valection with budgets increased a magnitude in size (n_targets_ = 1000, 2500, 5000, 10000, 25000). The budgets were, in this case, more proportionally similar to those of the synthetic dataset analysis when contrasted against the full known mutation set. However, the increased budget sizes have minimal effect on overall selection strategy performance and no effect on the relative strategy rankings (Additional file 1: Figure S13).

The second experimental dataset was obtained from Alioto et al. [23] and consists of a total of 15 somatic SNV call sets submitted by 14 teams, generated by running various calling algorithms on a single CLL tumour-normal sample. A gold set of verified SNV mutations was curated from these results and published, serving as the mutational ground truth. Valection was run on the samples with a slightly modified candidate budget size range (n_targets_ = 50, 100, 250, 500, 1000) due to there being a smaller set of known SNVs in this sample (*n* = 1319). Once again, results point to the ‘random rows’ method as the optimal selection strategy, with best overall performance and low spread in performance scores across submissions (Additional file 1: Figure S14).

## Discussion

Assessing and comparing the quality of new prediction tools is an important step in their adoption and the truth of their results is arguably the most important component of this assessment. When the resources required to independently verify results are substantial, it is vital to choose an unbiased but maximally informative set of results. This is naturally true not just for single-nucleotide mutations, but other predictions like structural variants, fusion proteins, alternative splicing events and epigenetic phenomena, e.g. methylation and histone marks. Ongoing research into the error profiles of various data types increases our understanding of what factors influence verification rates [24]. This information helps in distinguishing high- from low-quality calls and goes towards minimizing the amount of prediction verification required. However, with the continuous emergence of new data-generating technologies, e.g. third generation sequencing [25], benchmarking studies assessing false positive and false negative rates are likely to remain a fundamental component of computational biological research well into the foreseeable future. Having standardized methods for comparing workflows in contexts such as these will ease the uptake of new techniques more confidently. Valection is a first step towards standardizing and optimizing verification candidate selection.

Evaluation of the target candidate selection approaches presented in this study provides an in-depth view of the effects of call recurrence and algorithm representation on a verification candidate set. Nonetheless, this is by no means an exhaustive set of selection strategies. Although, our findings suggest that surprisingly straightforward approaches (e.g. ‘random rows’) are often the most effective, future implementations of more complex strategies may highlight additional factors important to target candidate selection. This is particularly true when error profiles are highly biased by known features of the dataset itself.

The need for informative verification target selections also highlights the importance of simulators for experimental biology, since the best suited method may vary from dataset to dataset. Indeed, as our findings here suggest, optimal candidate-selection strategies for mutation calls may even be affected by various tumour data characteristics. A complete assessment of error profiles is impossible without access to multifarious datasets with an established ground truth. As such, there is a need for reliable simulators in biology to create and analyze gold-standard synthetic datasets to help guide top empirical research. As demonstrated here, and specific to cancer genomics, synthetic tumour data can expedite accurate estimation of false negative rates which are difficult to determine in genome-wide mutation calling, mitigating the need for large-scale wet lab validation of non-variants. However, the utility of synthetic data is limited to non-exploratory research given that biological processes or data features that are unknown or poorly understood cannot be adequately simulated, leading to a lack of ‘real-world’ complexity. Therefore, the interplay between experimental and simulated data is critical to the advancement of disciplines such as genomics.

For these reasons, we included the evaluation of our software on ‘real’ data to determine the generalizability of our synthetic dataset analysis findings. It is key to note that the development of gold-standards from experimental data is fraught with its own set of biases. Validation experiments typically endeavour to use orthogonal sequencing technologies, which have largely independent error-profiles. However in practice, it is exceedingly rare for two technologies that measure a single phenomenon to be truly orthogonal. For example, DNA sequencing technologies typically exist down-stream of DNA extraction technologies, and thus share their biases. As another example, many sequencing techniques have challenges with repetitive regions (particularly homopolymer repeats), or lie up-stream of methods like sequence-alignment that have specific biases. Thus one key strategy to improving benchmarking is to rely on a battery of comparisons, with diverse gold-standards generated using both simulated and real data, and with the real data having a broad range of known biases that are clearly outlined to highlight potential correlations with the discovery data.

## Conclusions

Verification of somatic SNV calls made on NGS tumour data is critical due to the high numbers of false positive and false negative calls. However, a thorough search to identify all erroneous calls is a cumbersome and expensive task. Our findings suggest that it may also be an avoidable one. Fewer verification targets may be sufficient to characterize global error rates in data, provided that there is proper optimization of the target candidate selection process. We find that this optimization must factor in not just the scope of the verification study but, conceivably, the characteristics of the dataset itself. To date, few studies have assessed candidate-selection methods for verification purposes. Here, we begin to explore the alternatives available to genomicists performing confirmatory studies that are both efficient and thorough. By releasing our Valection software publicly, we encourage groups across the wider research community to continue this work. With a straightforward implementation and easy application, Valection has the potential for maximal impact across a wide range of disciplines that rely on verification studies.

## Methods

### Selection strategies & software

The random rows selection strategy (Fig. 1b) samples calls at random without replacement from the entire set of calls, and continues until the verification budget has been reached, or there are no more calls left.

The directed-sampling selection strategy (Fig. 1c) begins by constructing a matrix. Row 1 contains all the calls made only by individual callers, row 2 contains the calls made by exactly 2 callers, all the way to row N, which contains the calls that were made by all of the N callers. Each column, j, of the matrix contains only the calls made the j^th^ caller. Note that this means in all rows past 1, calls appear in multiple cells on the same row. Any given cell holds zero or more calls. To select calls, the following procedure is followed for each row, from N to 1, and for each cell in that row, ordered by ascending number of calls:

- Calculate the cell budget as the total remaining verification budget divided among the yet unexamined cells in the rest of the matrix.
- Select calls without replacement from the cell in question up to the cell budget (these calls become invalid selections for future cells). Each call selected reduces the total remaining verification budget.
- If any budget remains once all cells have been selected from, the process is repeated.

The equal per caller selection strategy (Fig. 1d) divides the verification budget equally among all callers. The set of calls that each individual caller made is sampled from without replacement up to that caller’s portion of the total budget. A call selected by one caller becomes an invalid choice for all other callers. If a single caller does not have enough available calls (calls not yet selected in another caller’s budget), its remaining budget is distributed equally to the other callers.

The equal per overlap selection strategy (Fig. 1e) is based around the number of times each call was made. With N callers, the verification budget is divided N ways. Out of the set of calls made only once (all the calls unique to any caller), calls are selected without replacement up to the sub-budget. This is repeated for all the calls made by exactly two callers, and so on up every level of overlap. If a single level of overlap does not have enough available calls (calls not yet selected in another overlap level’s budget), its remaining budget is distributed equally to the other levels.

The increasing with overlap selection strategy (Fig. 1f) is similar to equal per overlap, but instead of selecting an equal number of calls at every level of overlap, it selects a number from each level of overlap proportional to the level of overlap.

The decreasing with overlap selection strategy (Fig. 1g) is identical to increasing with overlap, but the number of calls selected at each level is inversely proportional to the level of overlap.

All of these methods are available through four commonly used programming languages C, Perl, Python and R. The implementations have robust user-level documentation and are openly available at both their appropriate public repositories (i.e. CPAN, PyPI and CRAN) and on our website at: labs.oicr.on.ca/boutros-lab/software/valection.

The selection strategy algorithms were implemented in C, and compiled using the GNU Compiler Collection (v4.8.1). The implementations also made use of GLib (v 2.44.0). The R statistical environment (v3.1.3) was used for statistical analysis and data subsetting. Perl (v5.18.2) was used to coordinate the simulations. All plots were generated with the same version of R using the “BPG” (v5.2.8) [26], “lattice” (v0.20–31) and “latticeExtra” (v0.6–26) packages. The analysis scripts are also available at http://labs.oicr.on.ca/boutros-lab/software/valection.

### Simulated data

To test the accuracy of these different approaches empirically, we applied them to gold-standard data from the ICGC-TCGA DREAM Somatic Mutation Calling Challenge [20]. This is a global crowd-sourced benchmarking competition aiming to define the optimal methods for the detection of somatic mutations from NGS-based whole-genome sequencing. The challenge has two components, one using simulated data created using BAMSurgeon software [4] and the other using experimentally-verified analyses of primary tumours. To test the accuracy of our approaches on representation algorithms, we exploited the SNV data from the first three in silico tumours. This dataset comprises 261 genome-wide prediction sets made by 21 teams and there are no access restrictions. The raw BAM files are available at SRA with IDs SRX570726, SRX1025978 and SRX1026041. Truth files are available as VCFs at https://www.synapse.org/#!Synapse:syn2177211**.** Prediction-by-submission matrices for all submissions are provided in Additional file 2: Table S1, Additional file 3: Table S2 and Additional file 4: Table S3, as well as the best submissions from each team in Additional file 5: Table S4, truth calls in Additional file 6: Table S5, Additional file 7: Table S6 and Additional file 8: Table S7 and a confusion matrix in Additional file 9: Table S8.

To probe a range of possible verification studies, we ran a very broad set of simulations. For each run, we pre-specified a tumour, a number of algorithms and a number of mutations to be selected for verification, and ran each of the candidate-selection strategies listed above. We then calculated the F_1_ score (along with precision and recall) based on the verification study, assuming verification results are ground truth. Finally, we compared the true F_1_ for a given algorithm on a given tumour across all mutations to the one inferred from the verification experiment.

We used three separate tumours with diverse characteristics (https://www.synapse.org/#!Synapse:syn312572/wiki/62018), including a range of tumour cellularities and the presence or absence of sub-clonal populations. We selected subsets of algorithms for benchmarking in four different ways:

i) the complete dataset (X)
ii) the single best submission from each team (X-best)
iii) three randomly selected entries from X-best (repeated 10 times)
iv) 25 randomly selected entries from X (repeated 10 times)

Lastly, we considered verification experiment sizes of 100, 250, 500, 1000 and 2500 candidates per tumour. Thus, in total, we analyzed each of the candidate-selection algorithms in 22 datasets for 3 tumours and 5 verification sizes, for 330 total comparisons.

### Experimental data

In addition to using synthetic data, we used two experimental datasets to thoroughly evaluate the Valection selection strategies. The first dataset consists of germline SNP information for the GIAB Consortium sample NA12878 [21, 22]. Germline mutation predictions were made on tissue samples sequenced on five platforms and analyzed using four SNP callers for a total of seven prediction sets. The second dataset comes from a mutation-calling benchmarking study that predicted somatic SNVs in a CLL tumour-normal sample [23]. This dataset comprises 15 somatic SNV prediction sets submitted by 14 teams. Information on the mutation predictions for these datasets is provided as Additional file 10: Table S9 and Additional file 11: Table S10.

As with the simulated dataset, we ran a number of simulations for each of our candidate-selection strategies with different combinations of the following two parameters: the number of algorithms/submissions sampled from and the number of mutations selected for verification (i.e. the candidate budget size). As before, we calculated the recall, precision and F_1_ score for each submission run and compared the true F_1_ for the submission to the verification experiment’s F_1_.

Because we had fewer prediction sets per tumour for the experimental datasets, we only ran two of the four previous algorithm subsets:

i) the complete dataset (X)
ii) 25 randomly selected entries from X

Regarding verification candidate budget sizes, for the first dataset (NA12878) we considered both the original set of sizes (n_targets_ = 100, 250, 500, 1000, 2500) as well as larger budget sizes, reflective of the ratio of verified germline mutations to somatic mutations (n_targets_ = 1000, 2500, 5000, 10000, 25000). For the second dataset (CLL), we only used smaller budget sizes since the data consists of somatic SNV calls. Given that the number of known somatic mutations for this dataset was 1319, the budget set size was modified not to exceed that amount (n_targets_ = 50, 100, 250, 500, 1000).

### Statistical analyses

The precision, recall and F_1_ score of each caller were calculated as follows, from the caller’s true positive (TP), false positive (FP) and false negative (FN) values, as estimated by the selection strategy. Here, FNs are true calls sampled by the selection strategy that were not made by the caller in question (i.e. another caller made it).

1 $$ precision=\frac{TP}{TP+ FP} $$

2 $$ recall=\frac{TP}{TP+ FN} $$

3 $$ {F}_1 score=2\kern0.5em \times \kern0.5em \frac{\left( precision\kern0.5em \times \kern0.5em recall\right)}{\left( precision\kern0.5em +\kern0.5em recall\right)} $$

When no calls were selected to calculate a value for a caller, scores were given values of N/A. This happened primarily with the ‘random rows’ method.

Additionally, each precision score was calculated in an adjusted and unadjusted manner. A caller’s precision in the unadjusted form was calculated exactly as described above, using all the calls made by the caller and selected for verification as the TPs and FPs. In the adjusted form, the selected calls were first divided into groups, according to how many callers made the call. Then, the precision was calculated separately using the calls from each group. The final precision was calculated as a weighted average of the precision of each group of calls, with weights equal to the total number of calls (verified and unverified) that caller made at that overlap level. Thus, in a two-caller example, a caller that made 100 unique calls and 50 calls shared with the other caller would count its precision from unique calls twice as strongly as its precision from shared calls.

## Availability and requirements

Project name: valection

Project home page: http://labs.oicr.on.ca/boutros-lab/software/valection

Operation Systems(s): any that support Perl, Python, R or C

Programming language: Perl, Python, R and C

License: GPL-3

## Additional files

Additional file 1: **Figure S1.** Simulations with 100 verification targets, across all synthetic tumours. Note: ‘random rows’ method generates N/As. **Figure S2.** All simulations with 1000 verification targets, across all synthetic tumours. **Figure S3.** All simulations with 2500 verification targets, across all synthetic tumours. **Figure S4.** All simulations with 250 verification targets, across all synthetic tumours. Note: ‘random rows’ method generates N/As. **Figure S5.** All simulations with 500 verification targets, across all synthetic tumours. Note: ‘random rows’ method generates N/As. **Figure S6.** All simulations for tumour IS1. Optimal results are achieved with the ‘equal per caller’ method (weighted mode). **Figure S7.** All simulations for tumour IS2. Optimal results are achieved with the ‘equal per caller’, ‘increasing per overlap’ and ‘equal per overlap’ methods (weighted mode). **Figure S8.** All simulations for tumour IS3. Optimal results are achieved with the ‘random rows’ method, regardless of how precision is calculated. **Figure S9.** a) Recall from all runs, displayed per candidate-selection strategy. b) Precision from all runs, calculated with and without a weight adjustment (default and weighted mode, respectively) and displayed per candidate-selection strategy. **Figure S10.** Replicate run scores for all synthetic data (a) and for a subset of 25 randomly selected submissions from that cohort (b). Selection strategies ordered by median scores. **Figure S11.** Replicate run scores for all the best synthetic data team submissions (a) and for a subset of 3 randomly selected submissions from that cohort (b). Selection strategies ordered by median scores. **Figure S12.** All simulations for sample NA12878 of the GIAB Consortium, with sampling budgets of 100, 250, 500, 1000, 2500. **Figure S13.** All simulations for sample NA12878 of the GIAB Consortium, with sampling budgets of 1000, 2500, 5000, 10000, 25000. **Figure S14.** All simulations for the CLL tumour-normal sample, with sampling budgets of 50, 100, 250, 500, 1000. (PDF 58025 kb)

Additional file 2: **Table S1.** A prediction-by-submission matrix of all SNV call submissions for tumour IS1 where SNV predictions are annotated with chromosome (“CHROM”) and position (“END”). (CSV 57526 kb)

Additional file 3: **Table S2.** A prediction-by-submission matrix of all SNV call submissions for tumour IS2 where SNV predictions are annotated with chromosome (“CHROM”) and position (“END”). (CSV 28680 kb)

Additional file 4: **Table S3.** A prediction-by-submission matrix of all SNV call submissions for tumour IS3 where SNV predictions are annotated with chromosome (“CHROM”) and position (“END”). (CSV 3656 kb)

Additional file 5: **Table S4.** A summary table of the top team submissions for each tumour, includes submission ID, team alias, the number of true positives, true negatives, false positives and false negatives, as well as the precision, recall and F_1_ scores. (CSV 3 kb)

Additional file 6: **Table S5.** A table of all predicted SNVs for tumour IS1, annotated by chromosome (“chrom”) and position (“pos”), and a “truth” column for whether the call is a true positive (1) or not (0). (CSV 3127 kb)

Additional file 7: **Table S6.** A table of all predicted SNVs for tumour IS2, annotated by chromosome (“chrom”) and position (“pos”), and a “truth” column for whether the call is a true positive (1) or not (0). (CSV 2537 kb)

Additional file 8: **Table S7.** A table of all predicted SNVs for tumour IS3, annotated by chromosome (“chrom”) and position (“pos”), and a “truth” column for whether the call is a true positive (1) or not (0). (CSV 328 kb)

Additional file 9: **Table S8.** A summary table of all submissions from across all synthetic tumours, includes submission ID, the number of true positives, true negatives, false positives and false negatives, as well as the precision, recall and F_1_ scores. (CSV 19 kb)

Additional file 10: **Table S9.** A summary table of all submissions from the first ‘real’ dataset, the GIAB sample NA12878, includes submission ID, the number of true positives, true negatives, false positives and false negatives, as well as the precision, recall and F_1_ scores. (CSV 694 bytes)

Additional file 11: **Table S10.** A summary table of all submissions from the second ‘real’ dataset, the CLL tumour-normal sample, includes submission ID, the number of true positives, true negatives, false positives and false negatives, as well as the precision, recall and F_1_ scores. (CSV 1 kb)

## Abbreviations

CLL: Chronic lymphocytic leukaemia
DREAM: Dialogue for reverse engineering assessments and methods
FN: False negative
FP: False positive
ICGC: International cancer genome consortium
NGS: Next-generation sequencing
SMC-DNA: Somatic Mutation Calling DNA Challenge
SNP: Single-nucleotide polymorphism
SNV: Single-nucleotide variant
TCGA: The cancer genome atlas
TP: True positive
