# Supplementary material for: Valection: design optimization for validation and verification studies
Source: BMC Bioinformatics. 2018 Sep 25;19:339. doi: 10.1186/s12859-018-2391-z (PMC6157051; doi:10.1186/s12859-018-2391-z)

Supplementary Figure 1

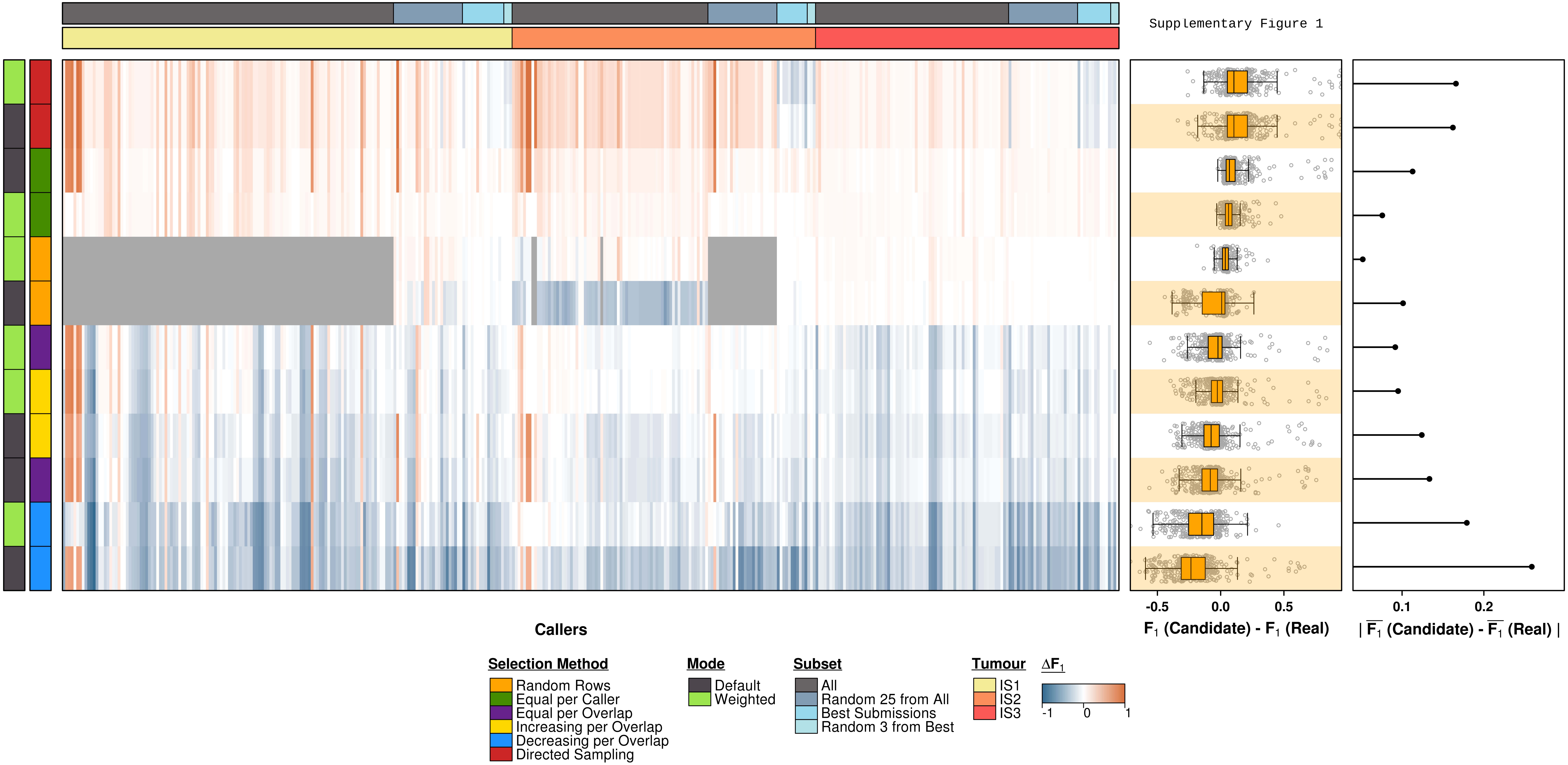

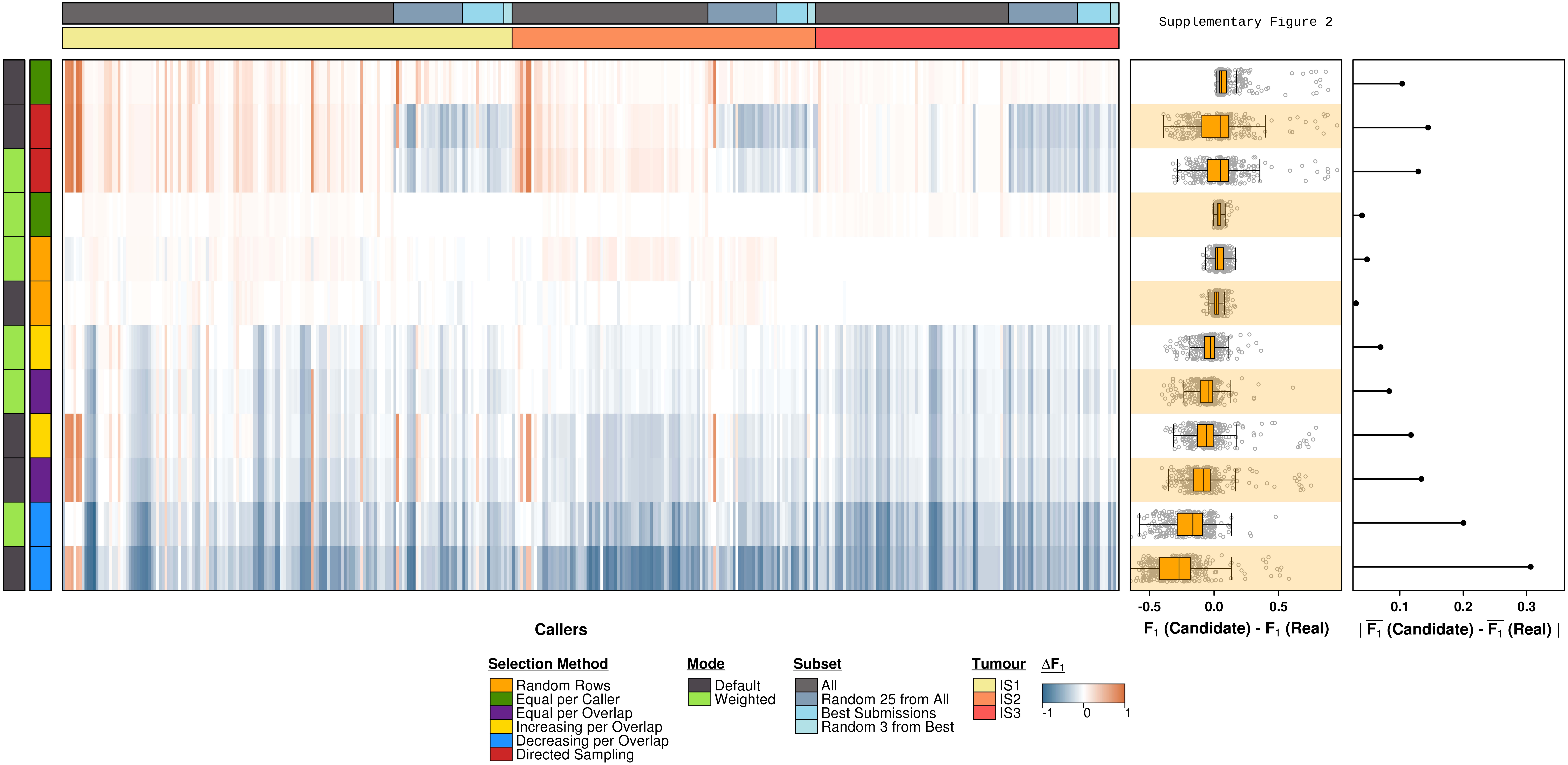
-0.5
0.0
0.5
F_1 (Candidate) - F_1 (Real)
0.1
0.2
0.3
| $\overline{F_1}$ (Candidate) - $\overline{F_1}$ (Real) |
Callers
Selection Method
Random Rows
Equal per Caller
Equal per Overlap
Increasing per Overlap
Decreasing per Overlap
Directed Sampling
Mode
Default
Weighted
Subset
All
Random 25 from All
Best Submissions
Random 3 from Best
Tumour
IS1
IS2
IS3
ΔF_1
-1
0
1

Supplementary Figure 3

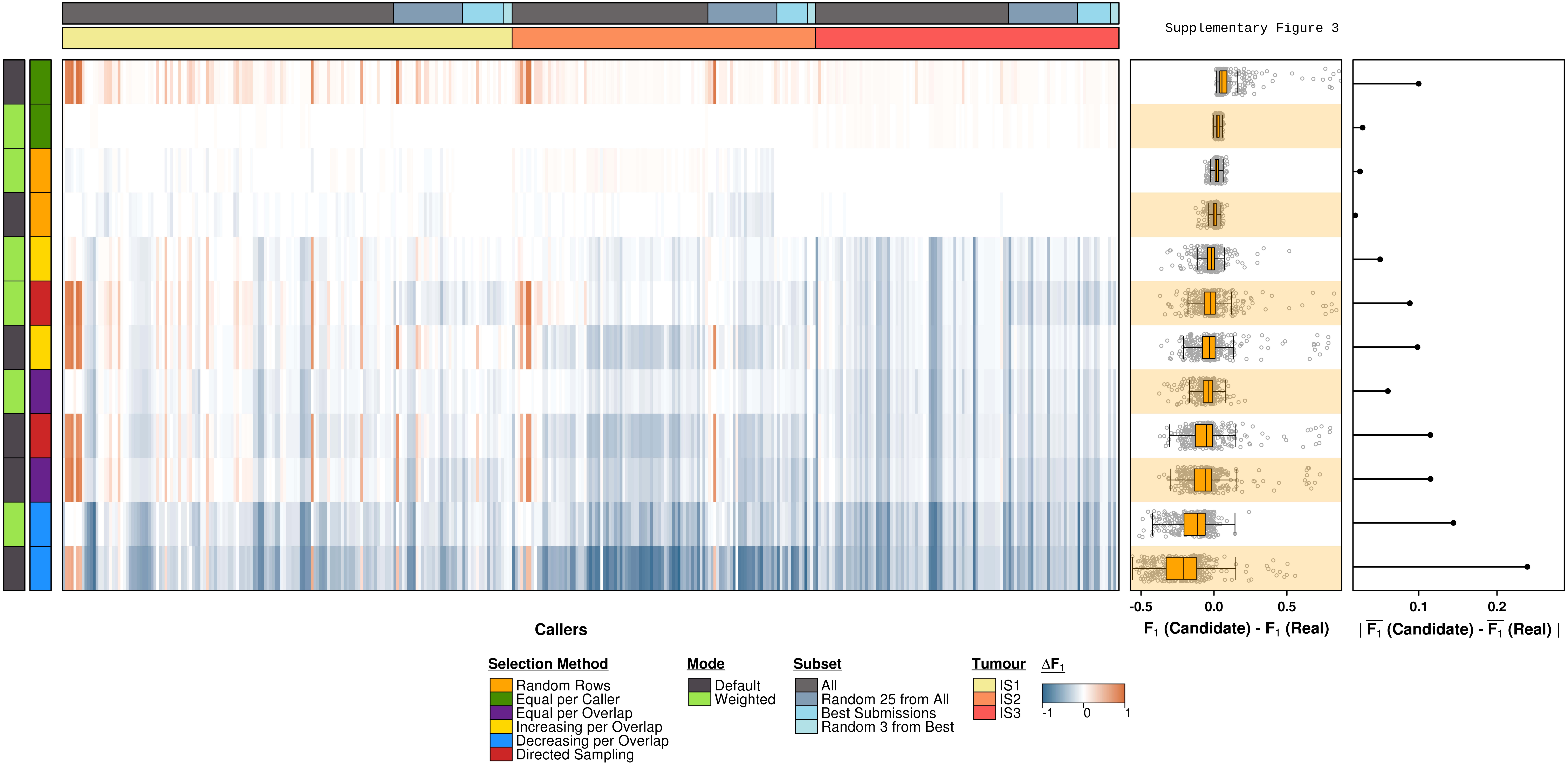

Supplementary Figure 4

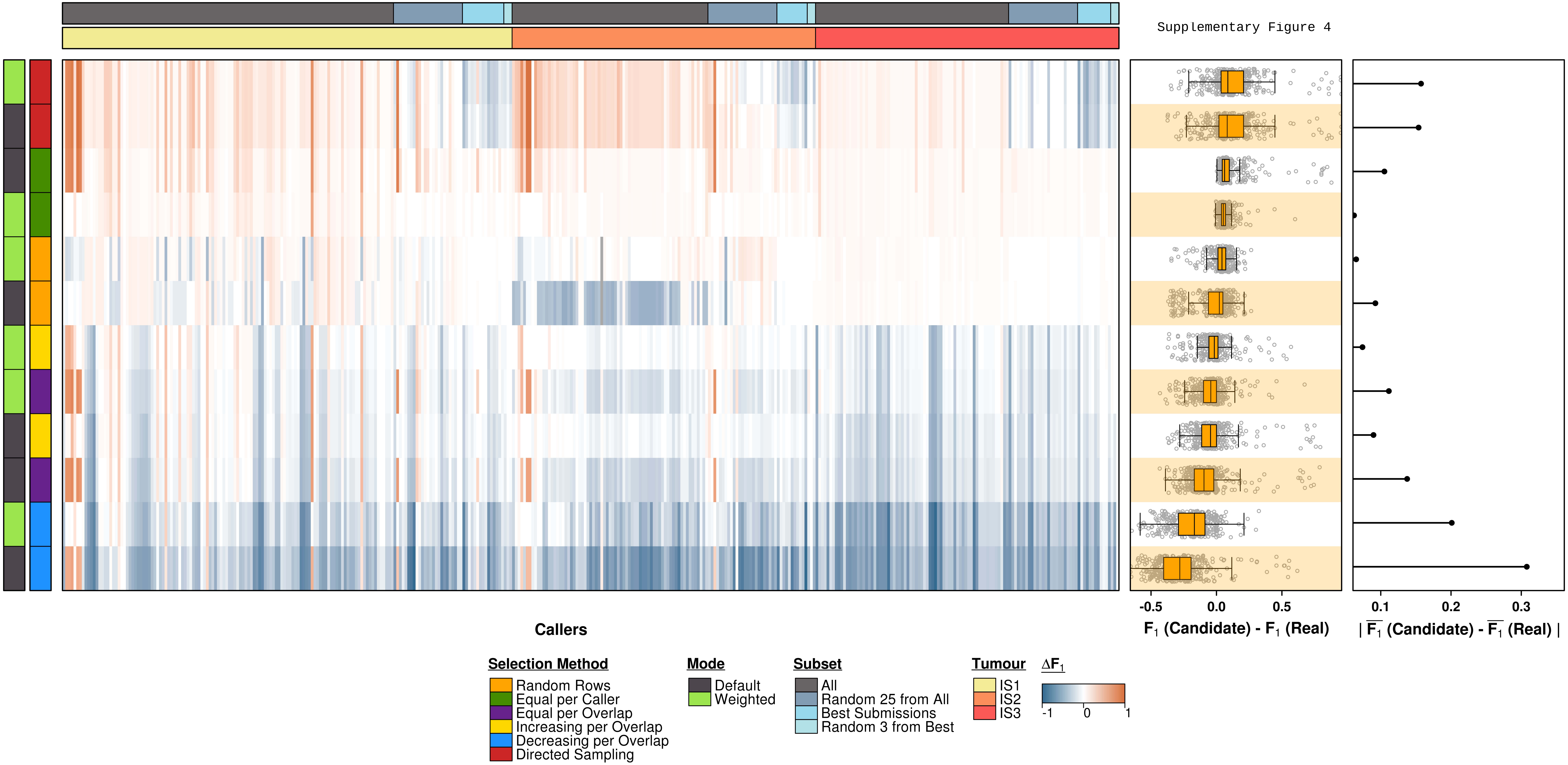

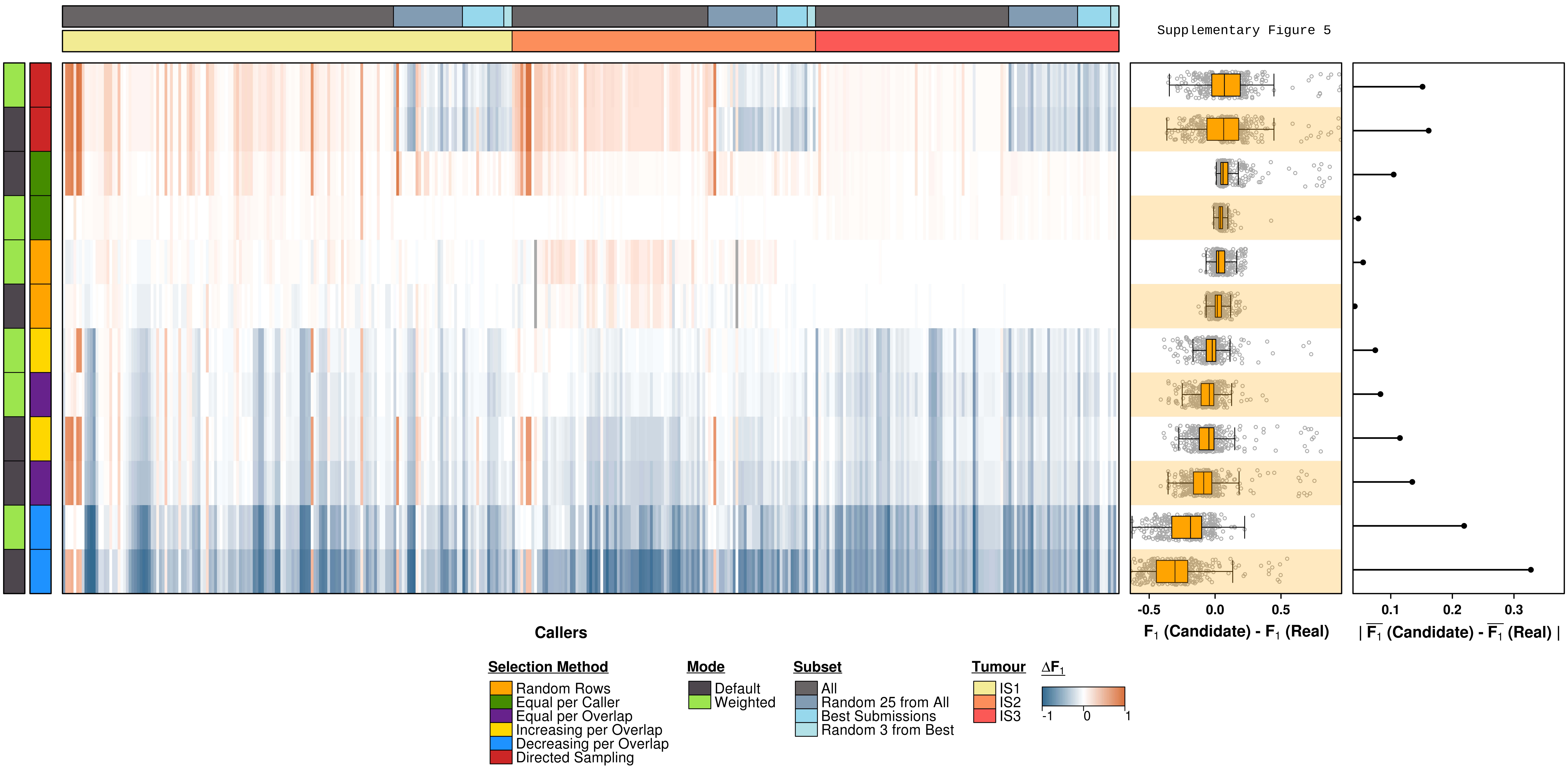

Supplementary Figure 5
Callers
-0.5
0.0
0.5
F_1 (Candidate) - F_1 (Real)
0.1
0.2
0.3
| $\overline{F_1}$ (Candidate) - $\overline{F_1}$ (Real) |
Selection Method
Random Rows
Equal per Caller
Equal per Overlap
Increasing per Overlap
Decreasing per Overlap
Directed Sampling
Mode
Default
Weighted
Subset
All
Random 25 from All
Best Submissions
Random 3 from Best
Tumour
IS1
IS2
IS3
ΔF_1
-1
0
1

Supplementary Figure 6

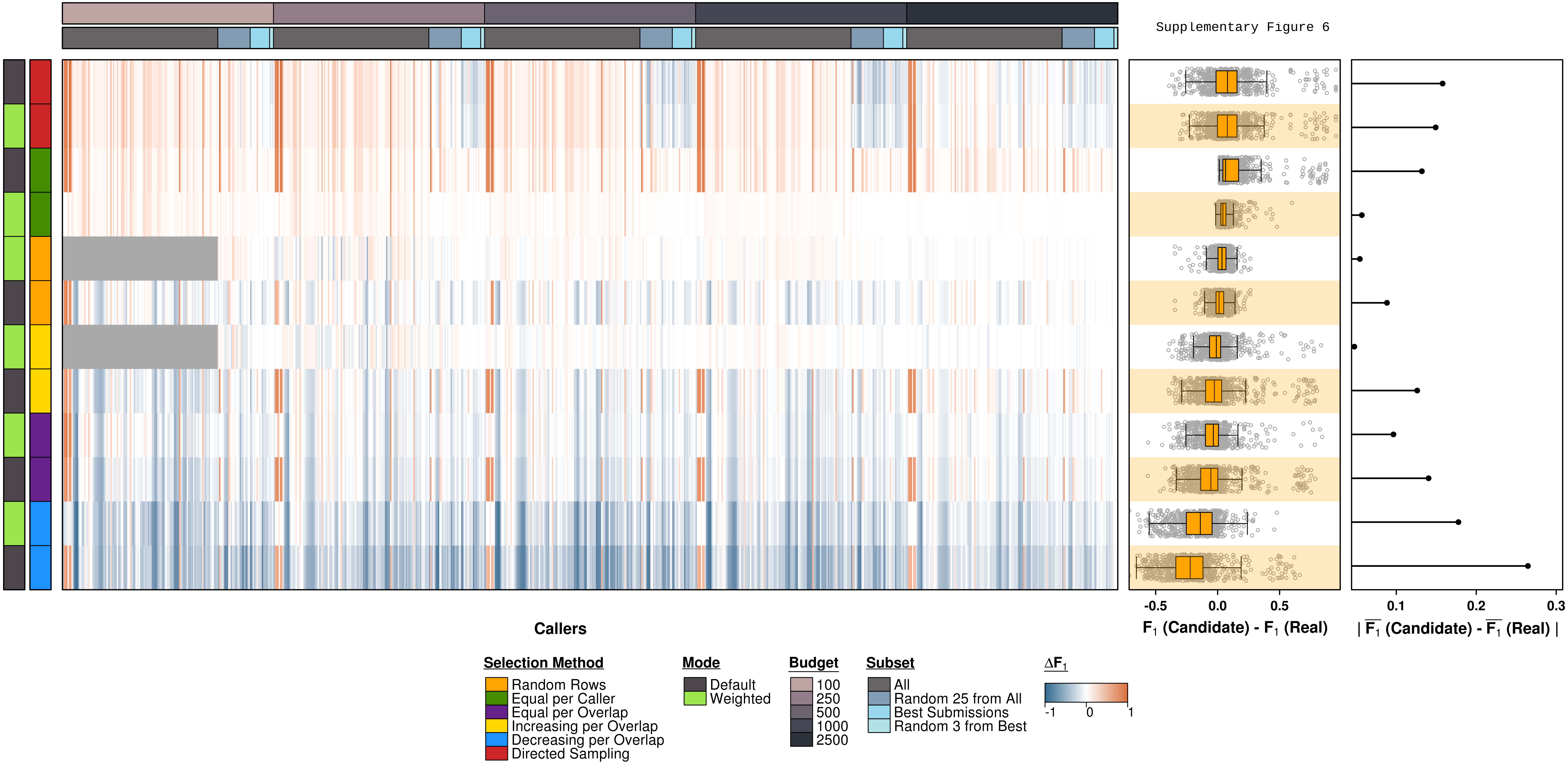

Supplementary Figure 7

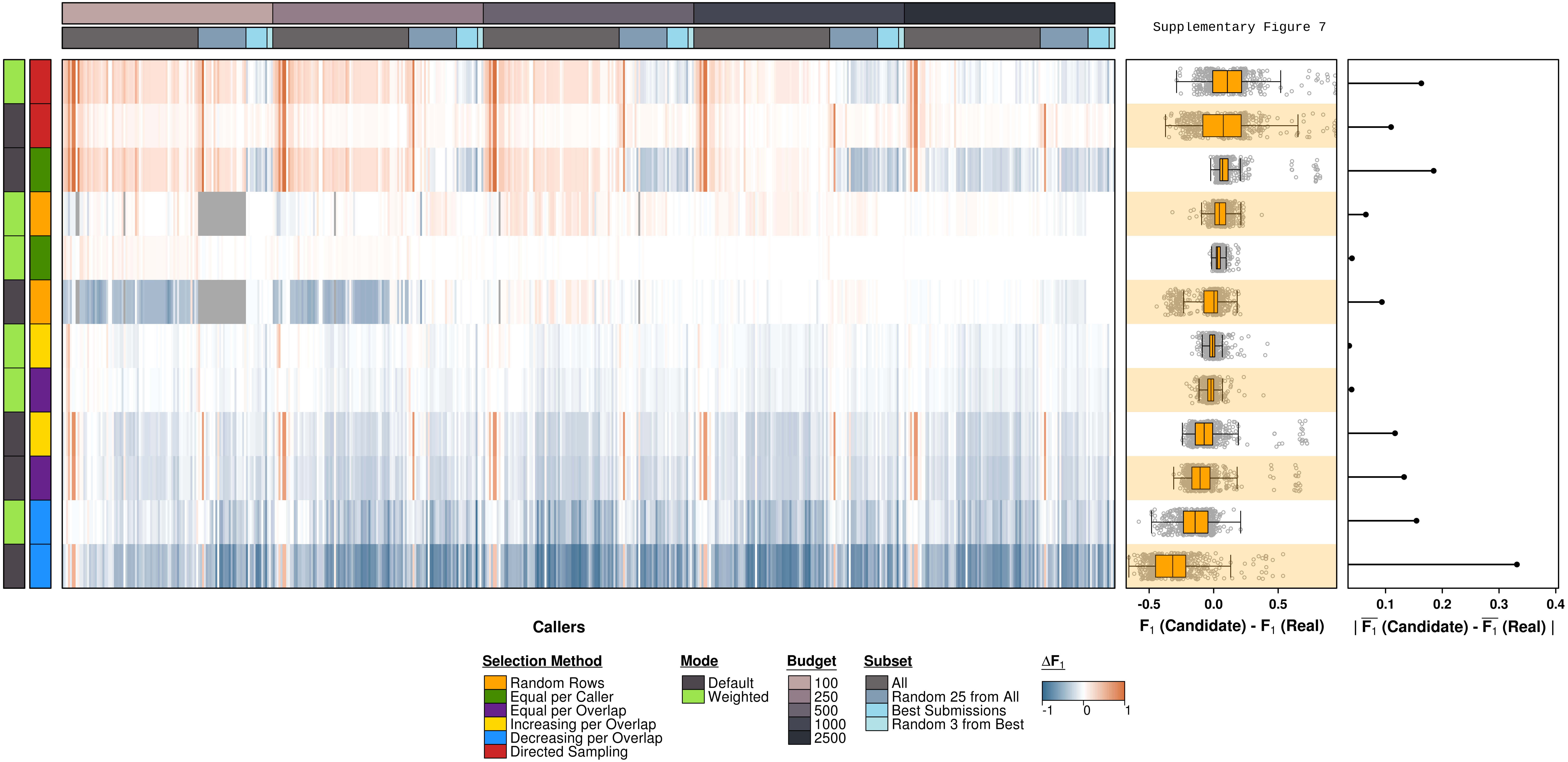

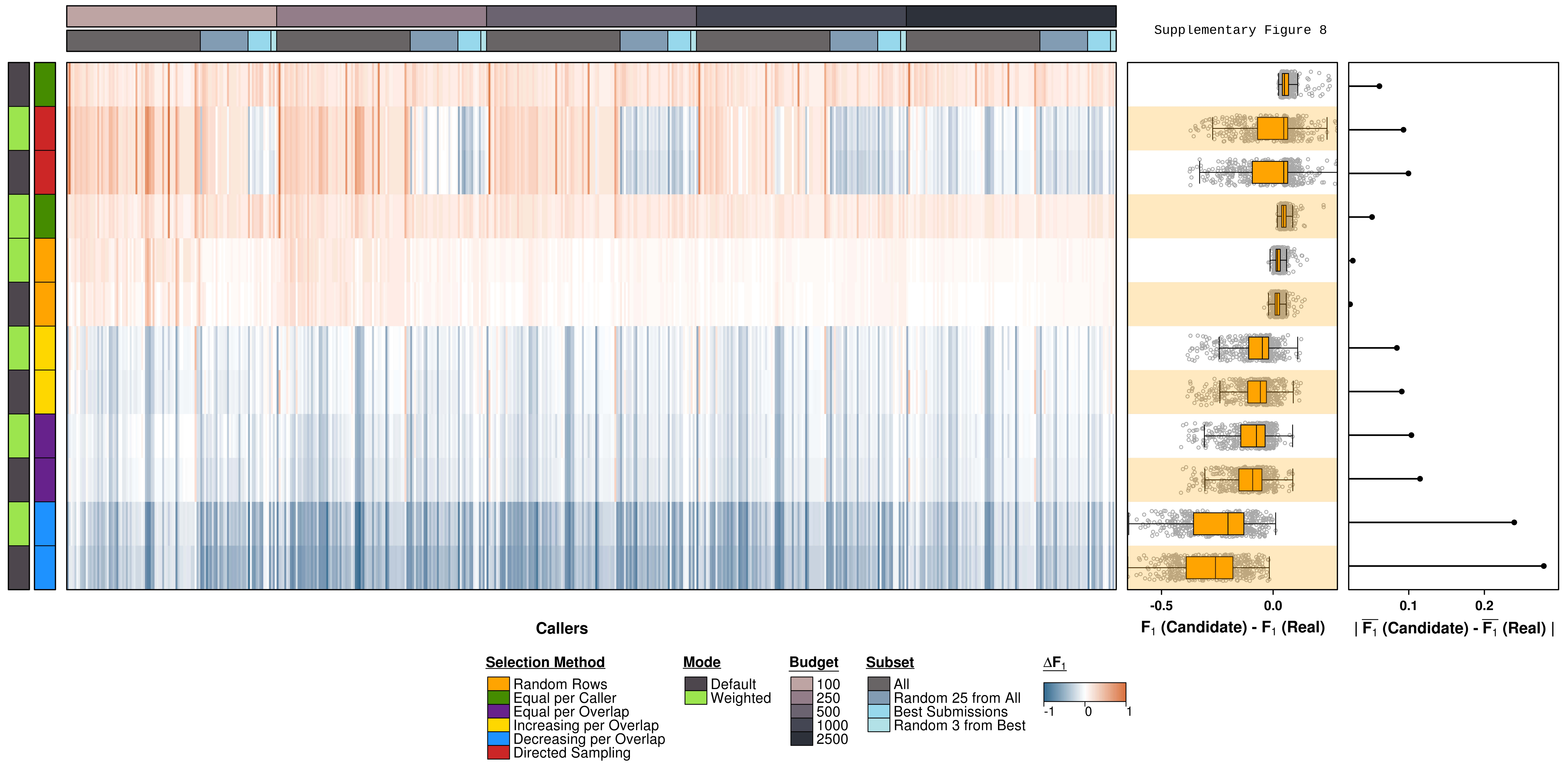
Supplementary Figure 8
-0.5
0.0
F1 (Candidate) - F1 (Real)
0.1
0.2
| F1 (Candidate) - F1 (Real) |
Callers
Selection Method
Random Rows
Equal per Caller
Equal per Overlap
Increasing per Overlap
Decreasing per Overlap
Directed Sampling
Mode
Default
Weighted
Budget
100
250
500
1000
2500
Subset
All
Random 25 from All
Best Submissions
Random 3 from Best
ΔF1
-1
0
1

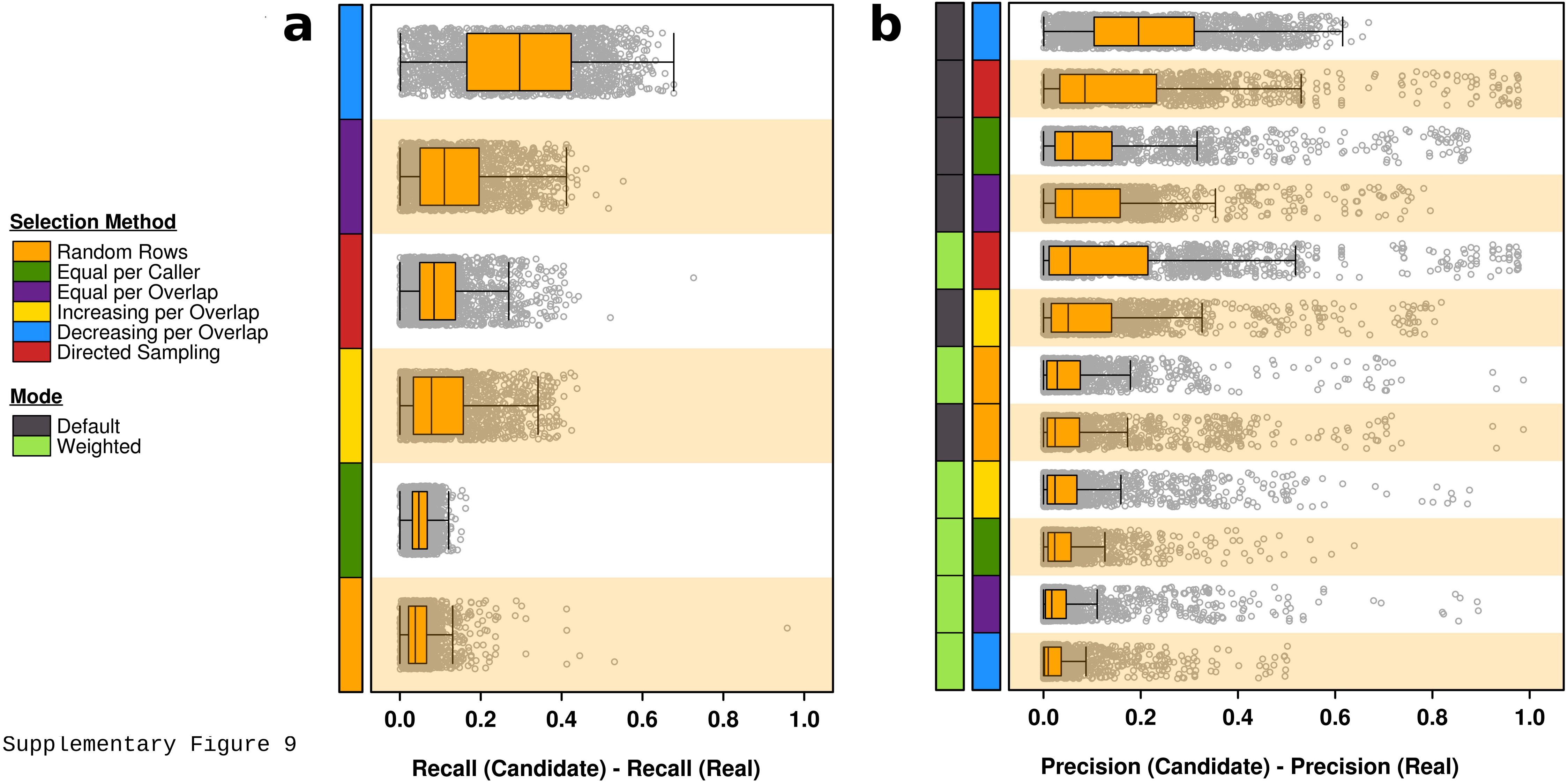

Supplementary Figure 9

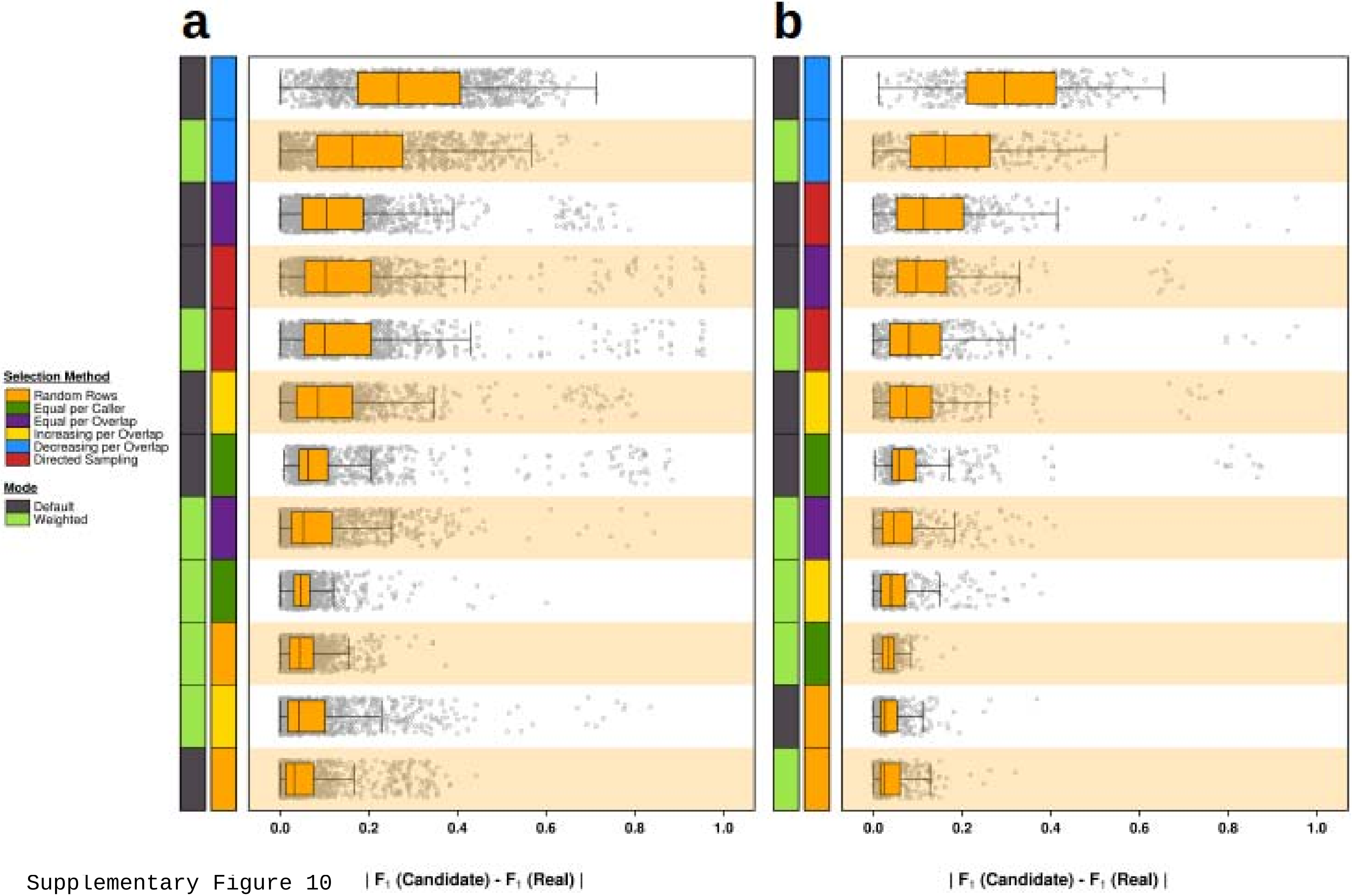

Supplementary Figure 10

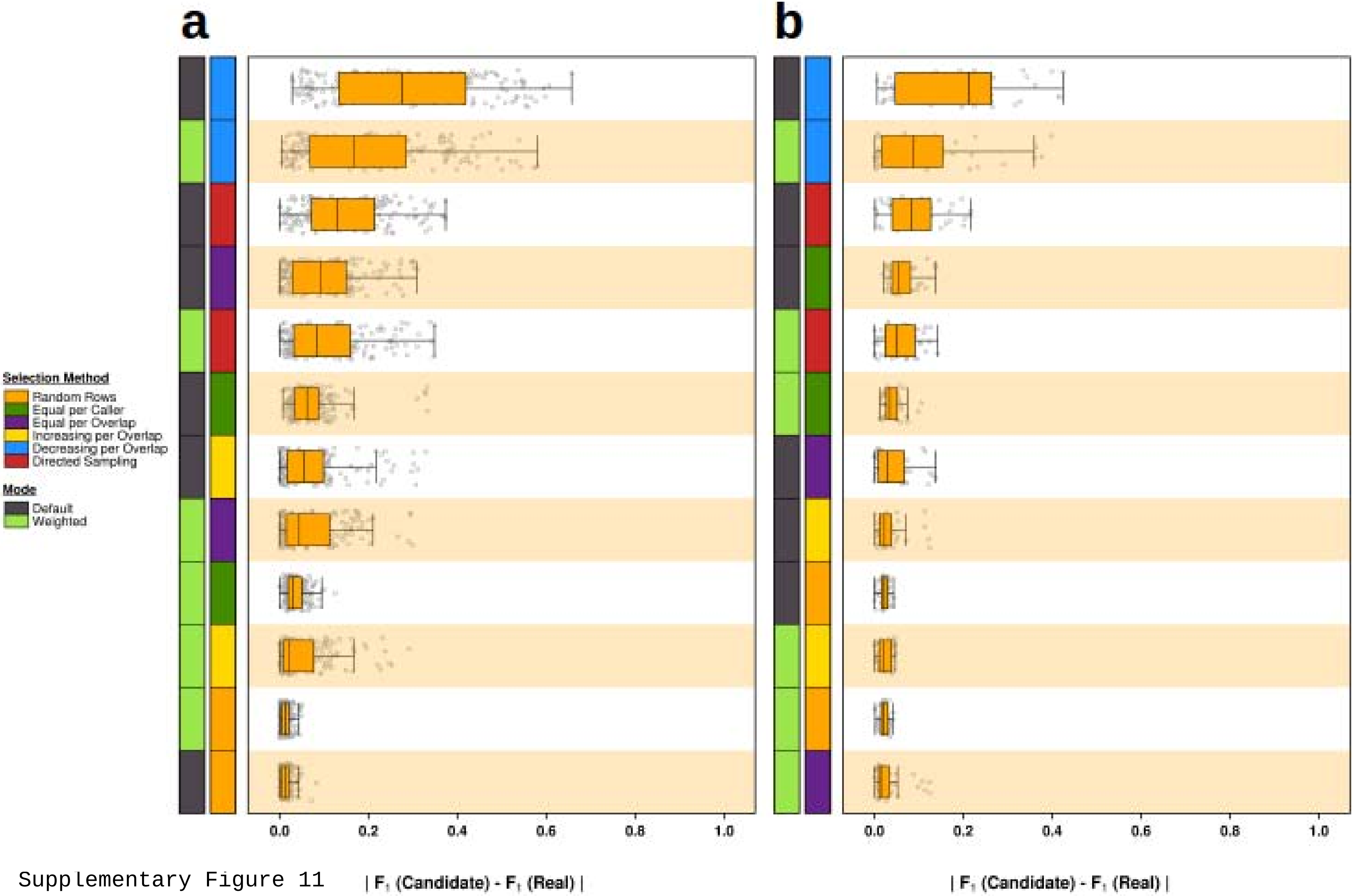

Supplementary Figure 11

Supplementary Figure 12

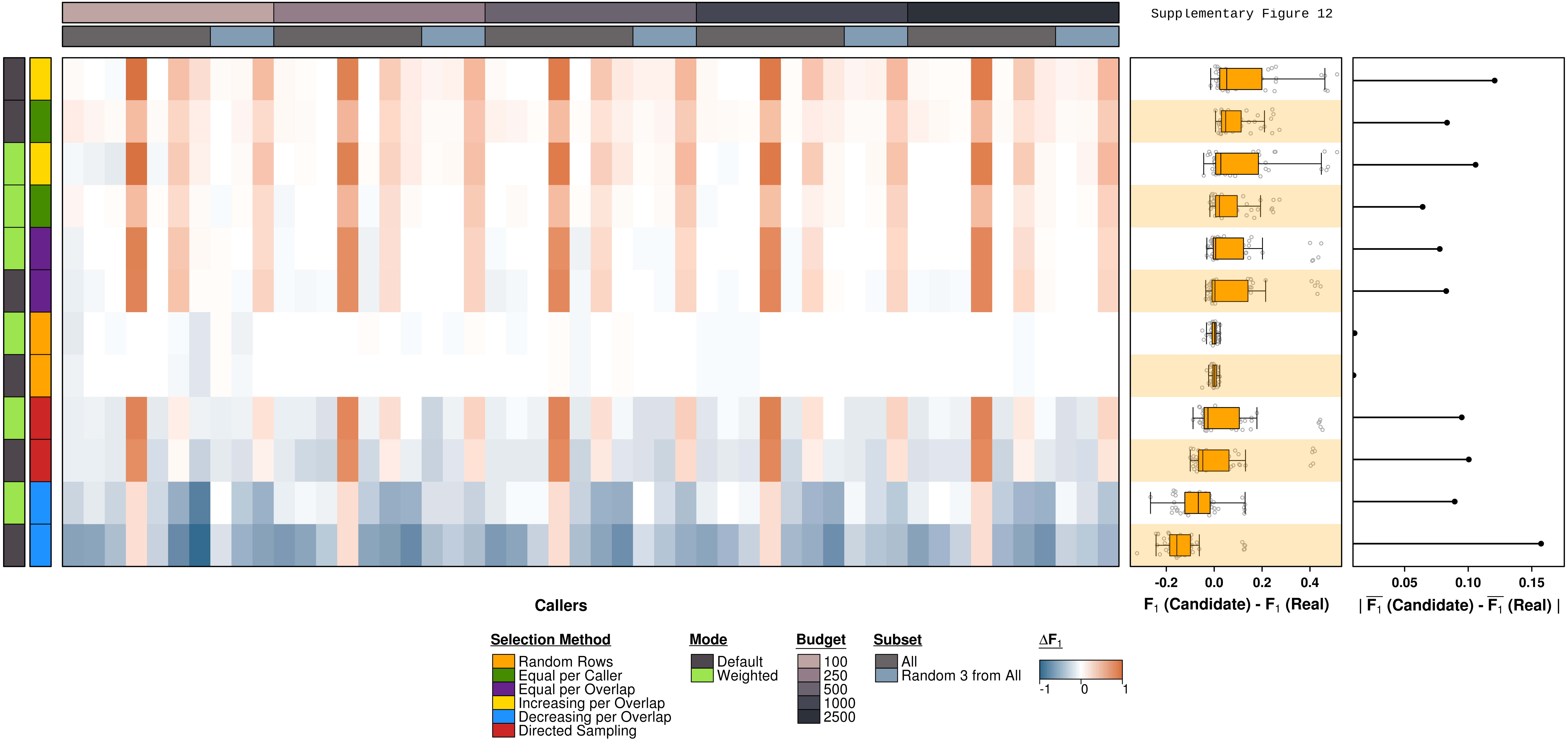

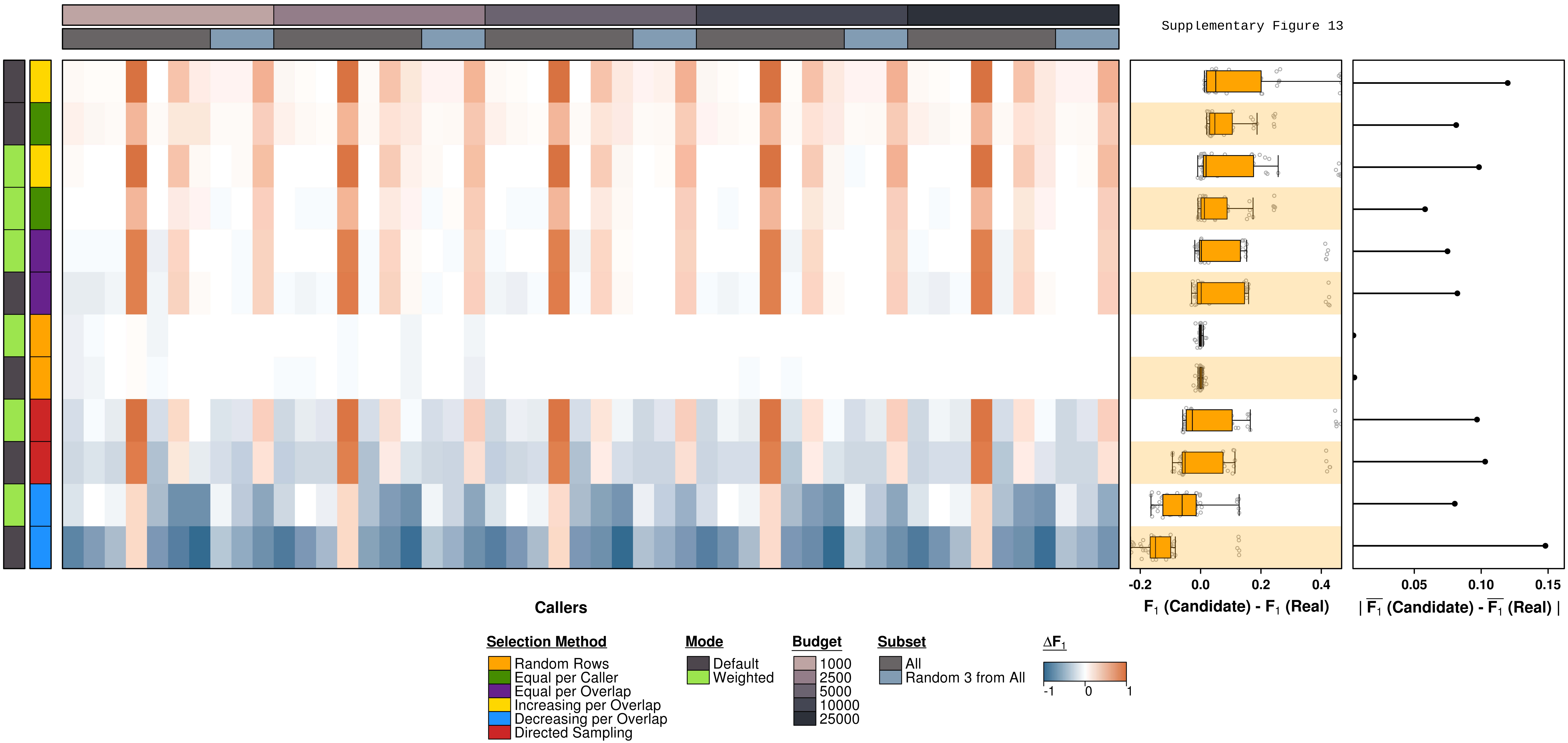

Supplementary Figure 13
Callers
-0.2
0.0
0.2
0.4
F1 (Candidate) - F1 (Real)
0.05
0.10
0.15
| F1 (Candidate) - F1 (Real) |
Selection Method
Random Rows
Equal per Caller
Equal per Overlap
Increasing per Overlap
Decreasing per Overlap
Directed Sampling
Mode
Default
Weighted
Budget
1000
2500
5000
10000
25000
Subset
All
Random 3 from All
ΔF1
-1
0
1

Supplementary Figure 14

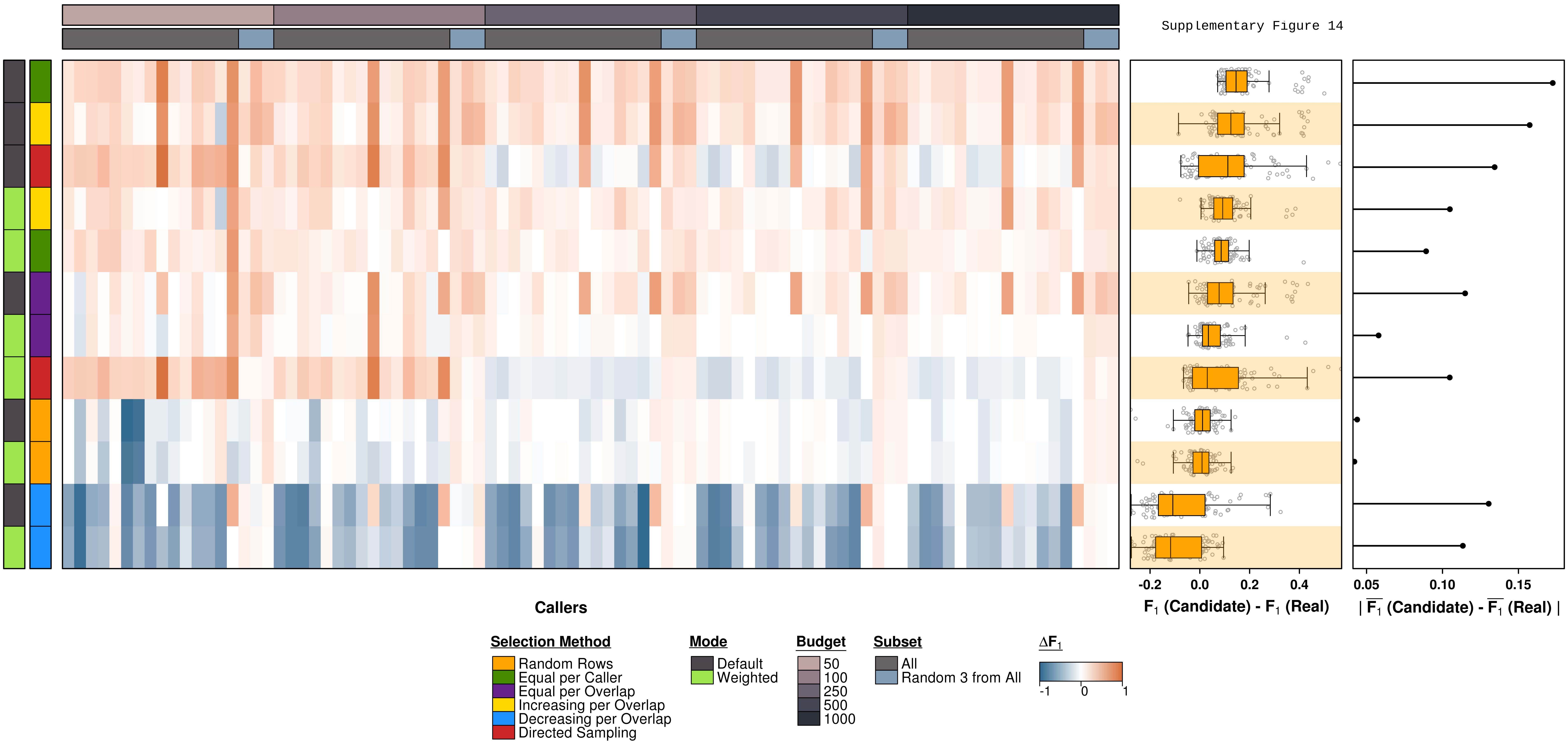

Supplement: Supplementary file 1 — Figure S1. Simulations with 100 verification targets, across all synthetic tumours. Note: ‘random rows’ method generates N/As. Figure S2. All simulations with 1000 verification targets, across all synthetic tumours. Figure S3. All simulations with 2500 verification targets, across all synthetic tumours. Figure S4. All simulations with 250 verification targets, across all synthetic tumours. Note: ‘random rows’ method generates N/As. Figure S5. All simulations with 500 verification targets, across all synthetic tumours. Note: ‘random rows’ method generates N/As. Figure S6. All simulations for tumour IS1. Optimal results are achieved with the ‘equal per caller’ method (weighted mode). Figure S7. All simulations for tumour IS2. Optimal results are achieved with the ‘equal per caller’, ‘increasing per overlap’ and ‘equal per overlap’ methods (weighted mode). Figure S8. All simulations for tumour IS3. Optimal results are achieved with the ‘random rows’ method, regardless of how precision is calculated. Figure S9. a) Recall from all runs, displayed per candidate-selection strategy. b) Precision from all runs, calculated with and without a weight adjustment (default and weighted mode, respectively) and displayed per candidate-selection strategy. Figure S10. Replicate run scores for all synthetic data (a) and for a subset of 25 randomly selected submissions from that cohort (b). Selection strategies ordered by median scores. Figure S11. Replicate run scores for all the best synthetic data team submissions (a) and for a subset of 3 randomly selected submissions from that cohort (b). Selection strategies ordered by median scores. Figure S12. All simulations for sample NA12878 of the GIAB Consortium, with sampling budgets of 100, 250, 500, 1000, 2500. Figure S13. All simulations for sample NA12878 of the GIAB Consortium, with sampling budgets of 1000, 2500, 5000, 10000, 25000. Figure S14. All simulations for the CLL tumour-normal sample, with sampling budgets of 50, 100, 250, 5 [file 12859_2018_2391_MOESM1_ESM.pdf]
